# Integrating Green Chemistry and Analytical Spectroscopy for Brilliant Blue G Removal Using Amberlite XAD7HP Resin

**DOI:** 10.3390/polym18141763

**Published:** 2026-07-19

**Authors:** Nicoleta Mirela Marin, Toma Galaon, Adriana Mariana Borș, Ludmila Motelica, Otilia Ruxandra Radacina, Marian Rascov, Ovidiu Oprea

**Affiliations:** 1 National Research and Development Institute for Industrial Ecology ECOIND, Street Podu Dambovitei no. 57-73, District 6, 060652 Bucharest, Romania; tomagalaon@yahoo.com; 2 Department of Analytical and Physical Chemistry, University of Bucharest, 4-12 Regina Elisabeta Bd., 030018 Bucharest, Romania; 3 Department of Oxide Materials Science and Engineering, National University of Science and Technology Politehnica Bucharest, 1–7 Gh. Polizu, 060042 Bucharest, Romania; otiliaradacina@yahoo.com (O.R.R.); marian.rascov@yahoo.com (M.R.); 4 National Institute for R&D for Optoelectronics—Subsidiary, Research Institute for Hydraulics and Pneumatics—INOE 2000-IHP, 040558 Bucharest, Romania; bors.ihp@fluidas.ro; 5 Research Center for Advanced Materials, Products and Processes, National University of Science and Technology POLITEHNICA Bucharest, Splaiul Independentei 313, 060042 Bucharest, Romania; ludmila.motelica@upb.ro; 6 National Centre for Micro- and Nanomaterials, National University of Science and Technology Politehnica Bucharest, 313 Independence Boulevard, 060042 Bucharest, Romania; ovidiu.oprea@upb.ro; 7 Academy of Romanian Scientists, 3 Ilfov St., 050045 Bucharest, Romania; 8 Faculty of Chemical Engineering and Biotechnologies, National University of Science and Technology POLITEHNICA Bucharest, Gh. Polizus 1-7, 011061 Bucharest, Romania

**Keywords:** Brilliant Blue G, polyacrylate resin, kinetics, adsorption isotherms, SEM/EDX morphology, FTIR-ATR spectroscopy, TG-DSC

## Abstract

This work presents an integrated green chemistry and analytical approach for the removal of Brilliant Blue G (BBG) using the non-ionic poly(acrylate) resin Amberlite XAD7HP (XAD7HP), emphasizing structure–property–performance relationships relevant to the resin’s adsorption behavior. The UV–Vis method used for BBG quantification exhibited excellent linearity in the 10–30 mg/L range (R^2^ = 0.9998). Batch adsorption experiments performed over 15–800 mg/L revealed a well-defined saturation profile, with the Langmuir model providing the best fit (R^2^ = 0.9999) and indicating a monolayer capacity of 117 mg/g and highly favorable adsorption (R_L_ = 0.001). Kinetic evaluation showed rapid initial uptake followed by intraparticle diffusion, with the pseudo-second-order model offering the highest correlation (R^2^ = 0.9811), while Weber–Morris analysis confirmed the contributions of both film and pore diffusion. FTIR-ATR analysis revealed only minor shifts (<10 cm^−1^) in characteristic bands, confirming physisorption driven by hydrogen bonding, π–π interactions, dipole–dipole forces, and hydrophobic effects, without structural modification of the resin. SEM/EDX imaging demonstrated significant morphological changes after adsorption, including partial pore blockage and deposition of dye aggregates within the meso–macroporous network. XRD patterns confirmed the structural stability of the resin, while TG-DSC analysis highlighted its thermal robustness and suitability for reuse. Desorption studies showed that acidic–alcoholic systems (MeOH–HCl, EtOH–HCl) ensured the highest BBG recovery, supporting the regenerability of XAD7HP. Overall, the combined spectroscopic, kinetic, equilibrium, and morphological evidence demonstrates that XAD7HP is a stable, efficient, and reusable resin for BBG removal, offering a sustainable remediation pathway aligned with green analytical chemistry principles.

## 1. Introduction

Currently, the rapid increase in global water consumption highlights the vulnerability of water systems and the need for integrated strategies for sustainable management [1,2,3,4]. International assessments indicate that by 2030, water consumption could exceed available resources by approximately 40% [5]. Furthermore, it is estimated that pressure on water resources will increase by up to 55% by 2050, primarily due to rapid urbanization and intensified industrial activities [6]. These trends threaten the stability of ecosystems and their capacity for self-regeneration, underscoring the need to strengthen interdisciplinary research and implement coherent public policies focused on resilience and sustainability [6,7,8,9,10].

Synthetic dyes are one of the most persistent and problematic classes of micropollutants in industrial wastewater, due to their chemical stability, resistance to biodegradation, and high ecotoxic potential [11,12]. BBG is widely used in the textile, food, bioanalytical, and biotechnology industries, and its presence in effluents leads to adverse effects on aquatic ecosystems, including the inhibition of photosynthesis, chronic toxicity, and bioaccumulation [13,14,15,16,17,18,19]. The extensive aromatic structure, sulfonate groups, and high molecular weight confer increased mobility in water and pronounced resistance to oxidative or biological processes to the molecule, which justifies the need for advanced and sustainable removal technologies [20,21,22]. In this context, adsorption has emerged as a versatile and effective technology for the removal of organic dyes, due to its low cost, regenerability, and adjustable selectivity [23,24]. Porous polymeric materials, particularly acrylic resins, have attracted increased interest due to their high specific surface area, chemical stability, and ability to interact with complex organic molecules through multiple mechanisms, such as hydrophobic interactions, hydrogen bonding, dipole–dipole forces, and π–π interactions [24,25,26,27,28,29,30,31,32]. Amberlite XAD7HP, a nonionic poly(acrylate)-based resin, exhibits a meso–macroporous architecture that facilitates intraparticle diffusion and allows for the establishment of complex physical interactions with bulky dyes such as BBG [33,34,35]. Previous research has explored various biosorbents and composite materials for the removal of Brilliant Blue G (BBG) from aqueous media. Several studies have reported adsorption capacities ranging from low to moderate values, depending on the nature and chemical modification of the sorbent. For example, dead biomass of the marine fungus Aspergillus wentii, in esterified, methylated, and raw forms, exhibited Langmuir monolayer capacities of 384.6, 370.4, and 312.5 mg/g, respectively [16]. Neodymium/alginate beads showed significantly lower BBG uptake (25.25 mg/g) under optimized conditions [36], while biocomposites based on Amberlite IRA resins and A. campestris achieved capacities of 149 and 142 mg/g at alkaline pH [37]. These studies highlight the variability of BBG adsorption performance across different materials and underline the need for further investigation of alternative sorbents with improved efficiency.

This study provides a novel and comprehensive investigation of BBG removal using the XAD7HP resin, a material that, to the authors’ knowledge, has not been previously evaluated for the adsorption of this dye. Unlike prior research [16,36,37,38,39,40], this study shows that XAD7HP—a commercially available, green, and regenerable resin—displays strong affinity toward BBG via hydrogen bonding, π–π interactions, dipole–dipole forces, and hydrophobic effects. The integration of FTIR-ATR spectral deconvolution with SEM/EDX morphological analysis provides mechanistic evidence that has not been reported before for resin–dye system. X-ray diffraction (XRD) analysis was also conducted to assess potential structural changes in the resin after BBG adsorption. Furthermore, the study establishes a robust kinetic and equilibrium modelling framework over an extended concentration range (15–800 mg/L). By combining green chemistry principles, advanced spectroscopic interpretation, and detailed mechanistic modelling, this work introduces a new sustainable pathway for the remediation of complex BBG from aqueous systems.

## 2. Materials and Methods

### 2.1. Chemicals

BBG, XAD7HP resin, 1 M hydrochloric acid (HCl), 1 M sodium hydroxide (NaOH), methanol (MeOH), and ethanol (EtOH) were purchased from Sigma-Aldrich (Merck, Darmstadt, Germany). The purification and conditioning of the XAD7HP resin were performed following the procedure described in our previous studies [20,26,41].

### 2.2. Linearity of Spectrophotometric Method

**Figure 1:**
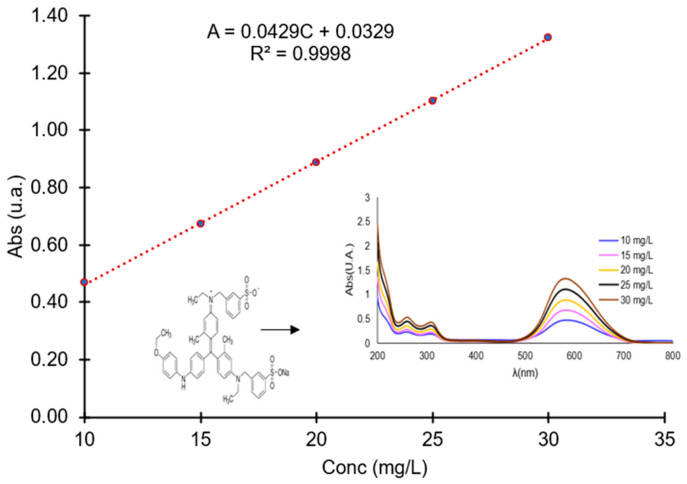
Linearity evaluation: UV–Vis absorption spectra and Beer–Lambert calibration plot at 585 nm of BBG.

The linearity of the spectrophotometric method was evaluated by constructing a calibration curve at λ = 585.0 nm using five standard solutions in the concentration range 10–30 mg/L. The corresponding absorbance values were 0.47, 0.67, 0.89, 1.10 and 1.33 for 10, 15, 20, 25 and 30 mg/L, respectively, showing a progressive increase in absorbance with concentration, in agreement with the Beer–Lambert law. A least-squares linear regression was performed by plotting absorbance (A) versus concentration (C, mg/L). The regression equation obtained was A = 0.0429 C + 0.0329 with a correlation coefficient R^2^ = 0.9998, indicating an excellent linear relationship between absorbance and concentration over the investigated range (Figure 1). The very high R^2^ value and the absence of any evident curvature in the calibration points confirm that the method is linear between 10 and 30 mg/L and suitable for quantitative determinations in this interval.

### 2.3. Procedure Applied for Evaluating the Kinetics of BBG Adsorption onto XAD7HP

The adsorption kinetics of BBG onto XAD7HP resin were evaluated under controlled batch conditions. Samples of XAD7HP (0.05 g) were accurately weighed and transferred into Erlenmeyer flasks. Subsequently, 0.01 L of BBG aqueous solutions with an initial concentration of 500 mg/L were added to the dry adsorbent. The mixtures were stirred at 175 rpm (T = 25 ± 2 °C) for 10, 20, 30, 40, 50, 60, 70, 80 and 90 min. At the end of each contact time, the mixtures were filtered, and the BBG concentration in the filtrate solutions was determined spectrophotometrically at λ = 585 nm. The quantity of BBG adsorbed at time t, Qt (mg/g), was calculated using Equation (1):

(1) $$Q_{t}=\frac{\left(C_{i}\;-\;C_{t}\right)V}{m}$$

where C_i_ is the initial concentration and C_t_ is the BBG concentration at time t (mg/L), V is the solution volume (L) of BBG, and m is the mass of XAD7HP resin (g).

### 2.4. Equilibrium Adsorption Procedure

Batch adsorption experiments were conducted to evaluate the uptake of BBG onto XAD7HP resin. BBG solutions C_i_ ranging from 15 to 800 mg/L were prepared in ultrapure water. For each adsorption experiment, 10 mL of dye solution was transferred into Erlenmeyer flasks, followed by the addition of ≈0.050 g of XAD7HP resin. The mixtures were placed in a horizontal shaker and maintained at T = 25 ± 2 °C at 175 rpm for 60 min. At end of stirring time, the resin was separated from the liquid phase by filtration, and the C_e_ was determined spectrophotometrically. The adsorption capacity at equilibrium (Qe, mg/g) of XAD7HP resin was calculated using Equation (2), while the removal percentage (R, %) was determined using Equation (3).

(2) $$Q_{e}=\frac{\left(C_{i}\;-\;C_{e}\right)V}{m}$$

(3) $$R\left(\%\right)=\frac{C_{i}-C_{e}}{C_{i}}\times 100$$

where C_e_ is the BBG equilibrium concentration (mg/L). The concentration range of 15–800 mg/L was selected to cover both environmentally relevant levels of BBG and the high-load conditions required to fully characterize the adsorption behavior of XAD7HP. Lower concentrations (15–150 mg/L) allow evaluation of the resin’s performance under typical wastewater scenarios, while higher concentrations (350–800 mg/L) are necessary to reach the saturation region. This wide range ensures a robust equilibrium dataset and enables reliable modeling of both the initial uptake region and saturation resin.

### 2.5. Desorption Procedure of BBG from XAD7HP Resin

The desorption of BBG from the XAD7HP resin was investigated using 1 M HCl, 1 M NaOH, MeOH, and EtOH, as well as the mixed systems 1:1 EtOH–1 M HCl and MeOH–1 M HCl. For this purpose, 0.5 g XAD7HP–BBG resin samples (loaded with 18 mg BBG/g resin) were accurately weighed into individual Erlenmeyer flasks and contacted with 0.05 L of each desorption agent. The resulting suspensions were stirred at 175 rpm for 30 min at T = 25 ± 2 °C. At the end of agitation time, the mixtures was separated by filtration through qualitative filter paper and the collected effluent solutions were analyzed spectrophotometrically.

The desorption percentage D(%) of BBG from the XAD7HP–BBG resin was quantified using Equation (4), which expresses the proportion of dye released into the desorption medium relative to the amount initially retained by the polymeric matrix.

(4) $$D(\%)=\frac{A}{B}\times 100$$

where A represents the mass of BBG (mg) released into the liquid phase during the desorption step, and B corresponds to the mass of BBG (mg) initially adsorbed onto the XAD7HP resin prior to desorption.

### 2.6. Fourier Transform Infrared Spectroscopy (FTIR-ATR)

A Nicolet iS50 FTIR spectrometer (Thermo Fisher Scientific Inc., Waltham, MA, USA), equipped with an ATR module and a DTGS detector, was used to record the spectra in the wavenumber range of 400 to 4000 cm^−1^. Each spectrum was obtained by averaging 32 scans at a resolution of 4 cm^−1^.

### 2.7. SEM/EDX Investigation

The surface morphology and microstructure of the films were investigated by scanning electron microscopy (SEM) using QUANTA INSPECT F50 equipment (FEI Company, Eindhoven, The Netherlands). The device is equipped with an energy-dispersive X-ray spectrometer (EDS) that was used to map the distribution of the elements in the samples.

### 2.8. X-Ray Diffraction (XRD)

The X-ray diffractograms were collected with a Rigaku device, model Miniflex 600 (Rigaku, Tokyo, Japan). Acquisition parameters were set as follows: voltage of 40 kV, current of 15 mA, scanning speed 1°/min, step size 0.01°, measurement range (2θ) 5–60°, copper Kα 1.5406 Å.

### 2.9. Thermogravimetry—Differential Scanning Calorimetry (TG-DSC)

A Netzsch STA 449 °C Jupiter device (Netzsch, Selb, Germany) was used for the thermal analysis (TG-DSC). The heating speed was 10 °C·min^−1^ up to 900 °C, under the flow of dried air at 50 mL/min.

## 3. Results and Discussion

### 3.1. Kinetics of BBG Adsorption onto XAD7HP

**Figure 2:**
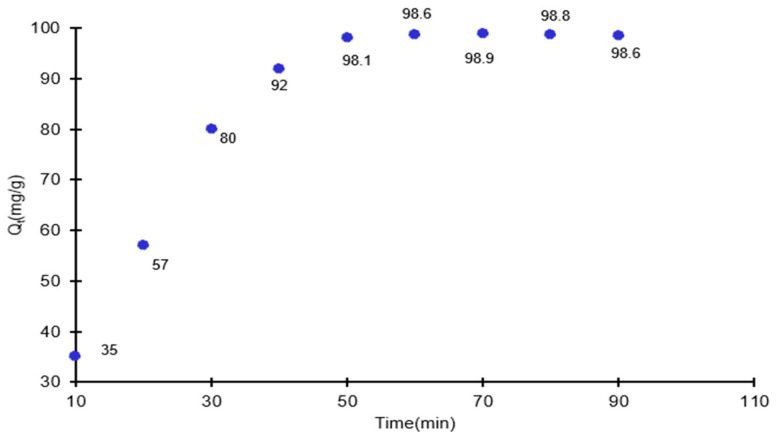
Evolution of Q_t_ (mg/g) during BBG adsorption onto XAD7HP over 10–90 min. Each reported value corresponds to the average of two independent measurements, both exhibiting standard deviations under 3%.

The adsorption behavior of BBG onto the XAD7HP resin was systematically evaluated over a contact time interval ranging from 10 to 90 min in order to elucidate the kinetic characteristics of the process (Figure 2). The obtained data reveal a clear multistage adsorption profile, typical for systems governed by both surface interactions and intraparticle diffusion phenomena.

At the initial stage from 0 to 10 min, the concentration of BBG in solution decreased from 500 mg/L to 325 mg/L, corresponding to Q_t_ = 35 mg/g. This rapid adsorption reflects the high affinity of the resin for BBG molecules and the abundance of readily accessible active sites on the polymer surface. This behavior is characteristic of diffusion-controlled processes where the driving force is the steep concentration gradient between the bulk solution and the resin surface.

Between 10 and 30 min, the Q_t_ values increased substantially from 35 mg/g to 80 mg/g, indicating that BBG molecules progressively migrated from the external surface into the internal porous network of XAD7HP. This stage is typically associated with intraparticle diffusion, where the rate of adsorption is influenced by the structural characteristics of the resin, including pore size distribution and internal surface accessibility. The continuous decrease in C_e_ during this interval confirms that the resin maintains a strong sorption potential as the dye penetrates deeper into the polymer matrix.

A further increase of time up to 40 min resulted a Q_t_ value of 92 mg/g, suggesting that the majority of the available adsorption sites had been occupied. Beyond this point, the adsorption rate slowed considerably. At 50 min, the adsorption capacity reached 98.1 mg/g, representing more than 98% removal efficiency. The subsequent measurements at 60, 70, 80, and 90 min showed only minimal variations (98.6–98.9 mg/g), indicating that the resin had reached its maximum Q_t_ value and that the system had stabilized.

The slight fluctuations observed at longer contact times (e.g., C_e_ values between 5.5 and 7 mg/L) are typical for systems operating near saturation, where minor desorption–readsorption events or analytical variability may occur. These variations do not significantly affect the overall adsorption performance and confirm that equilibrium was effectively established after approximately 60 min. The stabilization of Q_t_ values beyond this point demonstrates that the resin has fully expressed its adsorption capacity and that extending the contact time does not provide additional mechanistic insight or improvement in dye removal.

The kinetic profile obtained for BBG adsorption onto XAD7HP is consistent with the behavior reported for other polymeric adsorbents used for dye removal, where rapid initial uptake is followed by a slower diffusion-controlled phase [42,43,44,45,46,47,48]. The high adsorption capacity achieved within a relatively short time demonstrates the strong interaction between BBG and the hydrophobic–hydrogen bonding domains of the XAD7HP resin. Moreover, the near-complete removal of BBG from solution highlights the suitability of XAD7HP for applications involving high-concentration dye effluents.

To conclude, the results indicate that 60 min represents the optimal contact time for achieving equilibrium in the BBG–XAD7HP system. Extending the contact time beyond this point does not significantly enhance adsorption, confirming that the resin reaches saturation rapidly and maintains excellent retention efficiency throughout the experimental interval.

#### Kinetic Modeling

The kinetic analysis of the BBG–XAD7HP adsorption system was conducted to elucidate the rate-controlling mechanisms governing dye adsorption and to identify the steps involved in the overall mass transfer process. Kinetic models provide essential information regarding the interaction between the adsorbate and the adsorbent surface, the contribution of external and internal diffusion, and the temporal evolution of adsorption capacity.

To achieve a comprehensive understanding of the adsorption dynamics, the following kinetic models were evaluated: the pseudo-first-order (PFO), pseudo-second-order (PSO), Elovich, and intraparticle diffusion (Weber–Morris) models [44,48,49].

The mathematical expressions of these models (Equations (5)–(8)) allow for the determination of characteristic kinetic parameters that describe the adsorption rate, diffusion behavior, and the attainment of equilibrium.
Morris–Weber (intraparticle diffusion)(5)$Q_{t}=k_{id}{\left(t\right)}^{0.5}+C$Elovich(6)$Q_{t}=\ln(\alpha \beta )+\frac{1}{\beta }\mathrm{lnt}$PFO(7)$\log\left(Q_{e}\;-\;Q_{t}\right)=\log q_{e}\;-\;\left(\frac{k_{1}}{2.303}\right)t\;$PSO(8)$\frac{t}{Q_{t}}=\frac{1}{k_{2}Q_{e}2}+\frac{t}{Q_{e}}$
where k_id_ is a constant that expresses the rate at which BBG molecules diffuse into the internal pores of the XAD7HP resin. Higher values of k_id_ (mg/g min^−0.5^) indicate faster intraparticle transport and increased mobility of BBG within the polymer matrix. The parameter C represents the thickness of the external boundary layer surrounding the XAD7HP particles; a non-zero value of C confirms that intraparticle diffusion is not the only rate-limiting step. The constant α denotes the initial adsorption rate, reflecting the rapid absorption of BBG molecules onto the heterogeneous resin surface at the beginning of the process. The parameter β is associated with the degree of surface coverage and the activation energy required for continued adsorption; it describes the progressive decrease in the adsorption rate as the available sites on XAD7HP become increasingly occupied. The kinetic constants k_1_ and k_2_, corresponding to the pseudo-first-order (PFO) and pseudo-second-order (PSO) models, respectively, are used to evaluate the interaction mechanism between BBG and the resin surface. The parameter Q_e_ represents the equilibrium adsorption capacity, defined as the amount of dye retained per gram of XAD7HP once equilibrium is reached between the solid and liquid phases.

The linearized forms of the kinetic models are presented in Figure S2a–d of the Supplementary Materials, and the corresponding kinetic constants together with their correlation coefficients (R^2^), calculated based on these linear plots, are summarized in Table 1. The experimental equilibrium capacity was Qe, exp = 98.9 mg/g, which served as a benchmark for evaluating the predictive accuracy of each model.

Intraparticle diffusion (Morris–Weber) model showed the lowest correlation (R^2^ = 0.8197), indicating that pore diffusion is not the sole rate-limiting step. The non-zero intercept (C = 17.054 mg/g) confirms the presence of a boundary-layer effect, meaning that external film diffusion contributes significantly before intraparticle diffusion becomes relevant. Although intraparticle diffusion plays a role in the overall adsorption process, it cannot independently account for the observed kinetics.

The Elovich model provided a R^2^ = 0.9151, reflecting a heterogeneous adsorption surface and an increasing activation energy as adsorption progresses. The value of the initial adsorption rate (α = 5.03 mg/(g·min)) and the moderate desorption constant (β = 0.033 g/mg) indicate that the adsorbent surface contains energetically diverse sites.

The PFO model yielded a moderate correlation coefficient (R^2^ = 0.8895), indicating only partial agreement with the experimental data. However, the predicted equilibrium capacity (Q_e_, calc = 155.8 mg/g) substantially overestimated the experimental value (Q_e_, exp = 98.8 mg/g). This discrepancy between the PFO-calculated and experimental capacities clearly demonstrates that the PFO model is not suitable for describing the kinetic behavior of the system.

The PSO model exhibited the highest correlation coefficient (R^2^ = 0.9811), indicating a good fit to the experimental data. This strong correlation suggests that physisorption is the dominant rate-controlling mechanism. Nevertheless, the linearized PSO equation produced an unrealistically low equilibrium capacity (Q_e_, calc = 152 mg/g), far below Q_e_, exp. This discrepancy is a known limitation of the linearized PSO form, which can distort Q_e_ estimation in systems characterized by rapid initial uptake. Despite this limitation, the superior R^2^ and mechanistic relevance confirm that PSO best captures the kinetic trend, and Q_e_ should be interpreted based on experimental measurements rather than the linearized fit.

Overall, the combined kinetic evidence reveals a coherent mechanistic picture. The PSO model, which exhibits the highest R^2^ value, provides the best description of the adsorption kinetics. The Elovich model further supports this interpretation by indicating surface heterogeneity and a progressive increase in activation energy during adsorption. The Morris–Weber analysis confirms that both film diffusion and intraparticle diffusion contribute to the overall process, although neither governs the rate exclusively. In contrast, the PFO model is inconsistent with both the R^2^ values and the experimentally determined Q_e_.

### 3.2. Equilibrium Study of BBG Adsorption onto XAD7HP

**Figure 3:**
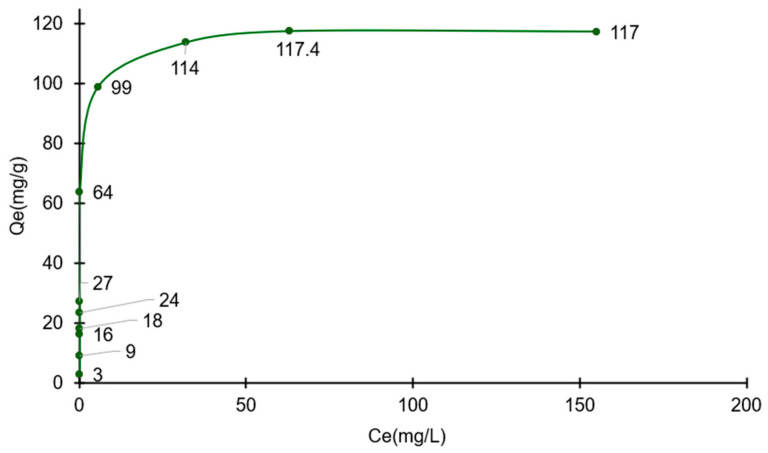
Experimental equilibrium isotherm illustrating the Q_e_ vs. C_e_ onto XAD7HP resin. Each reported value corresponds to the average of two independent measurements, both exhibiting standard deviations under 3%.

The adsorption behavior of BBG onto XAD7HP resin was evaluated across a wide range of initial concentrations (15–800 mg/L). The results reveal a clear and systematic evolution of the equilibrium concentration (C_e_) and adsorption capacity (Q_e_), reflecting the progressive occupation of available adsorption sites on the resin surface (Figure 3).

At low initial concentrations (15–50 mg/L), the equilibrium concentration remained extremely low, and the adsorption capacity increased from 3 to 9 mg/g. These values indicate that the resin surface provided a large number of readily accessible active sites, enabling near-quantitative removal of BBG from solution. The high uptake efficiency in this region demonstrates that mass transfer limitations were negligible and that the driving force for adsorption was strong.

For the moderate concentration range from 90 up to 150 mg/L, Q_e_ increased from 16 to 27 mg/g, confirming that the XAD7HP still possessed sufficient available sites to accommodate the increasing solute load. The continuous increase in adsorption capacity during this interval reflects efficient utilization of the resin surface and indicates that the system remained far from saturation.

A distinct transition toward surface saturation was observed at higher initial concentrations (350–500 mg/L). For this range, Q_e_ increased abruptly from 64 to 99 mg/g, while the equilibrium concentration rose noticeably, signaling a reduction in the number of unoccupied adsorption sites. Despite this shift, the resin maintained high adsorption performance, demonstrating strong affinity between BBG molecules and XAD7HP resin.

At the highest initial concentrations (600–800 mg/L), the system approached saturation. The equilibrium concentration increased substantially, while Q_e_ reached a plateau between 114 and 117 mg/g. This stabilization of adsorption capacity indicates that the available surface sites were nearly fully occupied. Across this concentration range, the removal efficiency remained high, varying between 80.6% and 94.7%, confirming that the resin continued to exhibit strong adsorption performance even under conditions of elevated solute loading.

Overall, the experimental data reveal a characteristic saturation profile, with rapid uptake at low concentrations, efficient adsorption across the intermediate range, and a well-defined plateau at high concentrations. This behavior is consistent with the progressive filling of a finite number of adsorption sites and provides a robust basis for subsequent isotherm modeling.

#### Adsorption Isotherm Modeling

The quantitative description of adsorption equilibrium is essential for understanding the mechanisms of interaction between adsorbate molecules and the surface of an adsorbent. Adsorption isotherms provide the mathematical basis for correlating the adsorption capacity at equilibrium (Q_e_) with the equilibrium concentration of the solute in solution (C_e_), allowing for the evaluation of surface properties, energy heterogeneity, and the nature of the adsorption process [50,51,52,53,54,55,56,57,58,59,60]. With these aims, the Langmuir (Equation (9)), Freundlich (Equation (10)), Temkin–Pyzhev (Equations (11) and (12)), and Dubinin–Radushkevich (D–R) (Equations (13)–(15)) isotherm models were applied to describe the adsorption of BBG on XAD7HP.

(9) $$\frac{C_{e}}{Q_{e}}=\frac{1}{bQ_{m}}+\frac{C_{e}}{Q_{0}}$$

(10) $$\ln Q_{e\;}=\ln K_{f}\;+\frac{1}{n}{\mathrm{lnC}}_{e}$$

(11) $$Q_{e}=\frac{\mathrm{RT}}{b_{T}}\ln\;(AC_{e})$$

and can be linearized as

(12) $$Q_{e}=\mathrm{BlnA}+\mathrm{Bln}C_{e}$$

(13) $$\ln Q_{e}=\ln q_{m}-\beta \varepsilon^{2}$$

(14) $$\varepsilon =\mathrm{RTln}(1+\frac{1}{C_{e}})$$

(15) $$E=\frac{1}{\sqrt{2\beta }}$$

where, for the Langmuir model, Q_0_ represents the theoretical monolayer adsorption capacity, while b expresses the affinity between BBG and the XAD7HP surface, and R_L_ evaluates the favorability of adsorption; the Temkin–Pyzhev constants A and b_T_ describe the adsorption energy distribution, with B reflecting the linear decrease in adsorption heat as surface coverage increases. In the Freundlich model, K_f_ denotes the adsorption capacity at low concentrations, whereas n and 1/n quantify surface heterogeneity and adsorption intensity. For the Dubinin–Radushkevich model, q_m_ represents the theoretical micropore filling capacity, β is related to the porosity and adsorption energy, and E indicates the mean adsorption energy, allowing discrimination between physical and chemical adsorption.

**Figure 4:**
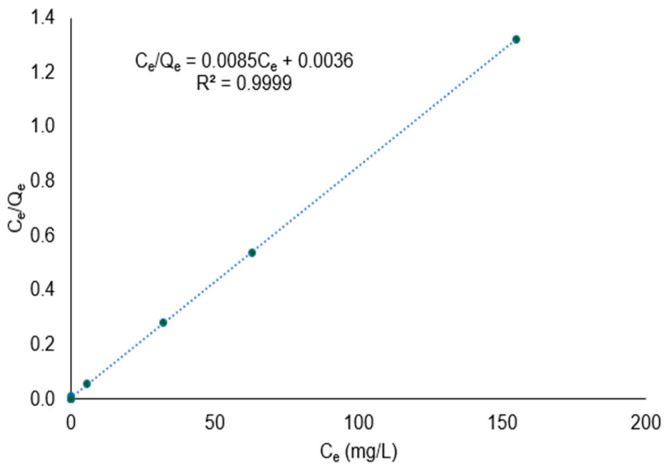
Linearized Langmuir adsorption isotherm for the BBG–XAD7HP system, expressed as the relationship between C_e_/Q_e_ and the equilibrium concentration C_e_.

The linearized form of the Langmuir isotherm is presented in Figure 4, while the linearized plots corresponding to the Freundlich, Temkin–Pyzhev, and D-R models are provided in the Supplementary Materials (Figure S1a–c) for completeness and comparative evaluation. On the basis of these linearized plots, the numerical values of the isotherm constants were obtained from the slopes and intercepts of the corresponding regression lines, and the resulting parameters of isotherm models are presented in Table 2.

The suitability of each isotherm model was assessed primarily through analysis of the R^2^ value, which represent the most robust statistical indicator for evaluating the agreement between experimental equilibrium data and the theoretical predictions of the models.

The Langmuir isotherm exhibited the highest degree of conformity with the experimental data, as reflected by the nearly perfect correlation coefficient (R^2^ = 0.9999). The Langmuir constants revealed a substantial monolayer adsorption capacity (Q_0_ = 117 mg/g) and a high affinity constant (b = 2.36 L/mg), while the extremely low separation factor (R_L_ = 0.001) confirmed that BBG adsorption onto XAD7HP is highly favorable. These results strongly support a monolayer adsorption mechanism occurring on a surface that behaves effectively as homogeneous.

In contrast, the Freundlich model provided a markedly poorer fit (R^2^ = 0.5906), indicating that the empirical multilayer description is not suitable for this system. Although the model yields a high Kf value (42.9 mg/g), the parameter 1/n = 0.25 suggests strong surface heterogeneity, which does not accurately represent the actual adsorption environment of the XAD7HP resin. The excellent Langmuir fit (R^2^ = 0.9999) confirms that adsorption occurs predominantly as a monolayer on uniform sites, and therefore the low 1/n value reflects the inadequacy of the Freundlich model rather than unfavorable adsorption.

The Temkin–Pyzhev isotherm yielded an intermediate correlation (R^2^ = 0.8839), implying that adsorbent–adsorbate interactions contribute to the adsorption process, but do not dominate it. The Temkin constants (A = 3.8 × 10^−2^ L/ mg, B = 11.5, b_T_ = 2.2 × 10^2^ J/mol) indicate a moderate interaction energy and a gradual decrease in adsorption energy with increasing surface coverage. Although this model captures certain energetic aspects of the system, its lower R^2^ relative to Langmuir demonstrates that it cannot fully describe the equilibrium behavior.

The D-R model showed the weakest agreement with the experimental data (R^2^ = 0.3094), confirming its limited applicability for quantitative interpretation. Nevertheless, the mean adsorption energy (E = 7.07 kJ/mol) falls within the characteristic range of physisorption, suggesting that the uptake of BBG onto XAD7HP is governed primarily by physical interactions such as van der Waals forces. The theoretical capacity predicted by the D–R model (q_m_ = 61 mg/g) is substantially lower than the Langmuir monolayer capacity, further supporting the inadequacy of this model for the present system.

Taken together, the comparative analysis clearly demonstrates that the Langmuir isotherm provides the most accurate and physically meaningful description of BBG adsorption onto XAD7HP. The results indicate a highly favorable, predominantly monolayer adsorption process driven by strong affinity and governed mainly by physical interactions.

### 3.3. Desorption Studies

**Figure 5:**
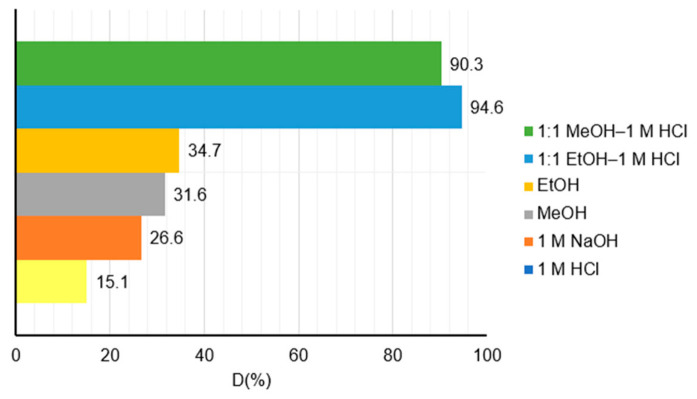
Influence of alcohol–acid mixtures and individual solvents on D(%) of BBG from XAD7HP. Each reported value corresponds to the average of two independent measurements, both exhibiting standard deviations under 3%.

The desorption behavior of BBG from the XAD7HP resin varied significantly depending on the nature of the desorbing agent, revealing clear differences in solvent–polymer and solvent–dye interactions (Figure 5) [20,31,41]. The weakest desorption efficiency was obtained with 1 M HCl, which released only 15.1% of the retained dye. This limited performance indicates that protonation of the dye and resin surface does not sufficiently disrupt the interactions responsible for BBG retention, suggesting that hydrophobic and π–π interactions are the dominant mechanisms stabilizing the adsorbed species.

A moderate increase in desorption efficiency was observed with 1 M NaOH (26.6%), reflecting the partial disruption of hydrogen bonding and possible deprotonation of functional groups. However, the relatively low value confirms that alkaline conditions alone are insufficient to overcome the strong affinity between BBG and the polymeric matrix.

The use of organic solvents produced higher desorption percentages, with MeOH (31.6%) and EtOH (34.7%) outperforming both acidic and alkaline aqueous media. This behavior highlights the contribution of hydrophobic and π–π interactions in the adsorption mechanism. EtOH exhibited slightly higher efficiency than MeOH, likely due to its greater ability to solvate aromatic moieties and weaken dye–resin interactions.

A dramatic enhancement in desorption was achieved when organic solvents were combined with acid. The mixed systems 1:1 EtOH–1M HCl and 1:1 MeOH–1M HCl yielded desorption efficiencies of 94.6% and 90.3%, respectively, demonstrating that the synergistic action of protonation (from HCl) and organic solvation (from alcohols) is highly effective in releasing BBG. Acidic conditions likely weaken hydrogen-bonding interactions, while the alcohol component disrupts hydrophobic and π–π stacking forces. The superior performance of the EtOH–HCl mixture compared to MeOH–HCl suggests that ethanol provides more efficient solvation of the aromatic dye structure, facilitating its transfer into the liquid phase.

Overall, the results indicate that BBG adsorption on XAD7HP is stabilized by a combination of hydrophobic, π–π, and secondary interactions, which cannot be effectively disrupted by aqueous acid or base alone. Only the combined action of an organic solvent and strong acid achieves near-quantitative desorption, confirming the strong affinity of BBG for the polymeric matrix and the necessity of mixed desorption systems for efficient resin regeneration.

### 3.4. FTIR-ATR Analysis

XAD7HP is a non-ionic microporous polymeric resin with a cross-linked poly(acrylate) structure, having a specific surface area of approximately 750 m^2^/g and an average pore diameter of ~550 Å [61]. Due to its aliphatic nature, this resin is capable of adsorbing both non-polar compounds from aqueous systems and polar compounds from non-polar solvents [62,63,64].

BBG (C.I. Food Blue 2, CAS 6104-59-2, MW = 854 g/mol) is a disulfonate triarylmethane dye featuring two -SO_3_^−^Na^+^ groups, tertiary amino groups, and extended aromatic rings [64,65]. Its presence in industrial wastewater poses an environmental issue, and treatment via adsorption on polymeric resins is an efficient approach [33,65,66].

FTIR spectroscopy is an essential method for characterizing dye adsorption on polymeric resins, enabling the identification of functional groups involved and elucidating interaction mechanisms [33,67,68,69,70].

#### 3.4.1. FTIR Spectrum of XAD7HP (Pure Resin)

**Figure 6:**
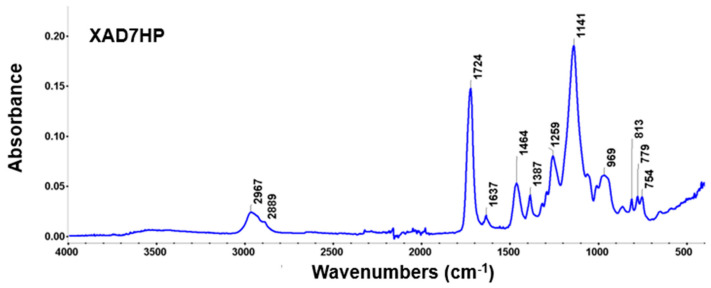
The FTIR spectrum of pure XAD7HP resin.

The FTIR spectrum of pure XAD7HP resin (Figure 6) reflects the characteristic structure of a cross-linked poly (n-butyl methacrylate). The identified bands are presented in Table 3.

The dominant band at 1141 cm^−1^, attributed to the symmetric stretching vibration ν(C–O–C) of the ester group, along with the strong carbonyl band at 1724 cm^−1^ [ν(C=O)], constitute the main spectroscopic markers of the polyacrylic structure [61,62,70]. These bands are in perfect agreement with data reported for poly(methacrylates) in the literature [70]. In the 2967–2889 cm^−1^ region, C-H stretching vibrations of methyl and methylene groups in the polymer chain appear, which is also confirmed by the deformation band at 1464 cm^−1^ [61,70]. The absence of any broad O-H (3200–3500 cm^−1^), N-H or S=O bands demonstrate the purity of the resin sample.

#### 3.4.2. FTIR Analysis of BBG (Pure Dye)

**Figure 7:**
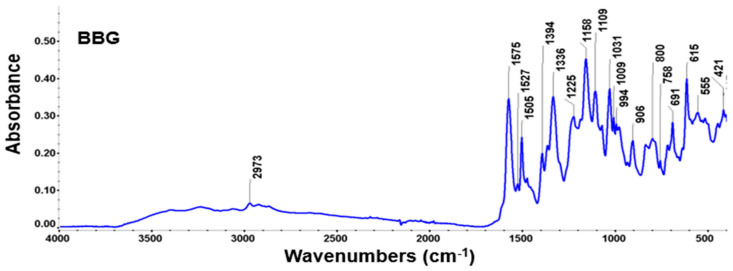
FTIR spectrum of pure BBG.

The FTIR spectrum of pure BBG (Figure 7) is more complicated than that of the resin, reflecting the structural complexity of the triarylmethane sulfonate molecule [64,65,71].

Absorption bands are concentrated in the 400–1700 cm^−1^ range, whereas C-H bands in the 3000 cm^−1^ region are weakly represented due to the predominance of rigid aromatic fragments over aliphatic chains [64,65,71]. Identified bands are listed in Table 4.

Aromatic band clusters at 1575–1505 cm^−1^, attributed to ν(C=C) vibrations of the conjugated triarylmethane skeleton, are characteristic of this dye class [64,65]. The sulfonate doublet at 1158/1109 cm^−1^ [ν_as_(S=O)] represents the definitive spectroscopic markers of the -SO_3_^−^ groups, confirming the disulfonate structure of BBG [65,73]. Symmetric frequency (ν_s_) of S=O bands at 1031–994 cm^−1^ complete the spectral fingerprint of the sulfonate groups [65,73]. Multiple out-of-plane γ(C–H) bands of aromatic protons appear in the 900–691 cm^−1^ domain, characteristic of 1,2,4-trisubstituted and monosubstituted rings in the BBG structure [64,72].

#### 3.4.3. FTIR Analysis of XAD7HP-BBG

**Figure 8:**
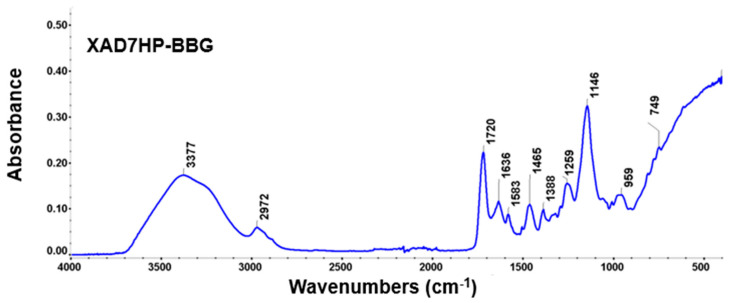
FTIR spectrum of the XAD7HP-BBG.

The FTIR spectrum of the XAD7HP-BBG (Figure 8) is analytically the most significant, as it reveals the structural changes induced by dye adsorption onto the resin, offering insights into the interaction mechanism. To support clearer visualization of the spectral modifications associated with BBG adsorption, the overlaid FTIR spectra of the native and BBG-loaded XAD7HP resin have been included in the Supplementary Material in Figure S3. Also, the bands are compared side-by-side in Table 5.

#### 3.4.4. New Bands Appearing After Adsorption

The most notable change in the BBG spectrum relative to the pure precursors is the emergence of a broad, intense band at 3377 cm^−1^, assigned to ν (O–H) and ν (N–H) stretching vibrations.

This band, completely absent in both pure XAD7HP and pure BBG, indicates the formation of intermolecular hydrogen bonds between the hydroxyl/amino groups of the dye and the adsorption centers of the resin, alongside the incorporation of water molecules into the adsorbed BBG [63,66,69]. The appearance of such broad bands post-adsorption aligns with similar studies on dye adsorption over polymer resins [33,63,66]. Another new band relative to pure XAD7HP occurs at 1583 cm^−1^, shifted by 8 cm^−1^ compared to its position in the pure BBG spectrum (1575 cm^−1^). This is attributed to the aromatic ν(C=C) vibration of the adsorbed dye [33,64,66]. This blue shift points to a perturbation of the π-electrons within the BBG aromatic rings due to interactions with the resin surface.

#### 3.4.5. Spectral Shifts and Intensity Modifications

The resin’s carbonyl band ν(C=O) shifts from 1724 cm^−1^ (pure XAD7HP) to 1720 cm^−1^ (BBG), a red shift of −4 cm^−1^. This shift demonstrates a weakening of the C=O bond, likely caused by dipole–dipole interactions with the sulfonate groups of BBG [61,63,66].

The dominant esteric νs (C–O–C) band shifts from 1141 cm^−1^ to 1146 cm^−1^ (+5 cm^−1^), accompanied by a minor intensity increase. This implies a partial overlap with the ν(S=O) bands from the BBG spectrum, along with an environmental chemical perturbation of the ester group [61,65,66]. The band at 1636 cm^−1^ exhibits higher intensity than in pure XAD7HP, reflecting contributions from the aromatic rings of the adsorbed BBG [33,66].

#### 3.4.6. Diminished or Missing Bands

The characteristic sulfonate doublet of pure BBG (1158/1109 cm^−1^) is strongly attenuated in the complex spectrum. This is potentially due to the participation of -SO_3_^−^ groups in interactions with the resin surface and overlap with the (C–O–C) bands of the polymer matrix [63,65,66,73]. The dense aromatic bands of BBG (1505–1527 cm^−1^) are also visibly reduced in intensity and resolution within the complex [33,64,66].

#### 3.4.7. Adsorption Mechanism—FTIR Interpretation

Comparative analysis of the three FTIR spectra provides clear spectroscopic evidence regarding the adsorption mechanism of BBG onto XAD7HP resin [33,37,63,66].

Physical nature of adsorption (physisorption): The absence of any new chemical bond bands (no new amide, sulfonamide, ester, or other covalent group bands are formed in the spectrum of the complex) demonstrates that the adsorption of BBG onto XAD7HP is predominantly physical and reversible—physisorption [68,69,70]. This conclusion is supported by the small shifts of existing bands (<10 cm^−1^) instead of the disappearance or appearance of new chemical bonding bands.

#### 3.4.8. Involved Interactions

Based on the identified spectral changes, the following physical interactions can be proposed:

(i) π-π forces: the shift of the aromatic ν(C=C) band of BBG from 1575 cm^−1^ to 1583 cm^−1^ indicates a perturbation of the dye’s π-electrons from the aromatic rings through interaction with the polymeric matrix.

XAD7HP, although aliphatic, can mediate hydrophobic and disperse-type interactions involving the π systems of BBG [73,74].

(ii) Dipole–Dipole/van der Waals interactions: the shift of the ν(C=O) band from 1724 cm^−1^ to 1720 cm^−1^ suggests an interaction between the ester carbonyl of the resin and the sulfonate groups of BBG, likely mediated by induced dipole–dipole forces [63,66,68].

(iii) Hydrogen bonds: the emergence of the broad band at 3377 cm^−1^ indicates the formation of hydrogen bonds, possibly between the -OH/-NH groups of the dye and the electronegative centers of the resin, or via co-adsorbed water molecules [63,66,69].

(iv) Hydrophobic interactions: the non-ionic and aliphatic nature of XAD7HP favors the adsorption of compounds with a partial hydrophobic or amphiphilic character, such as BBG with its bulky aromatic rings [37,61,74,75].

### 3.5. SEM/EDX Analysis

#### 3.5.1. SEM Analysis

**Figure 9:**
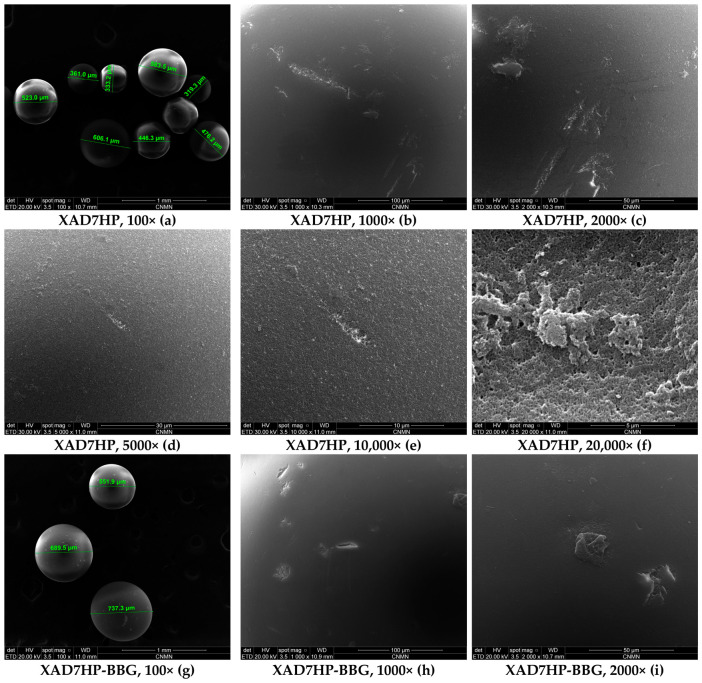

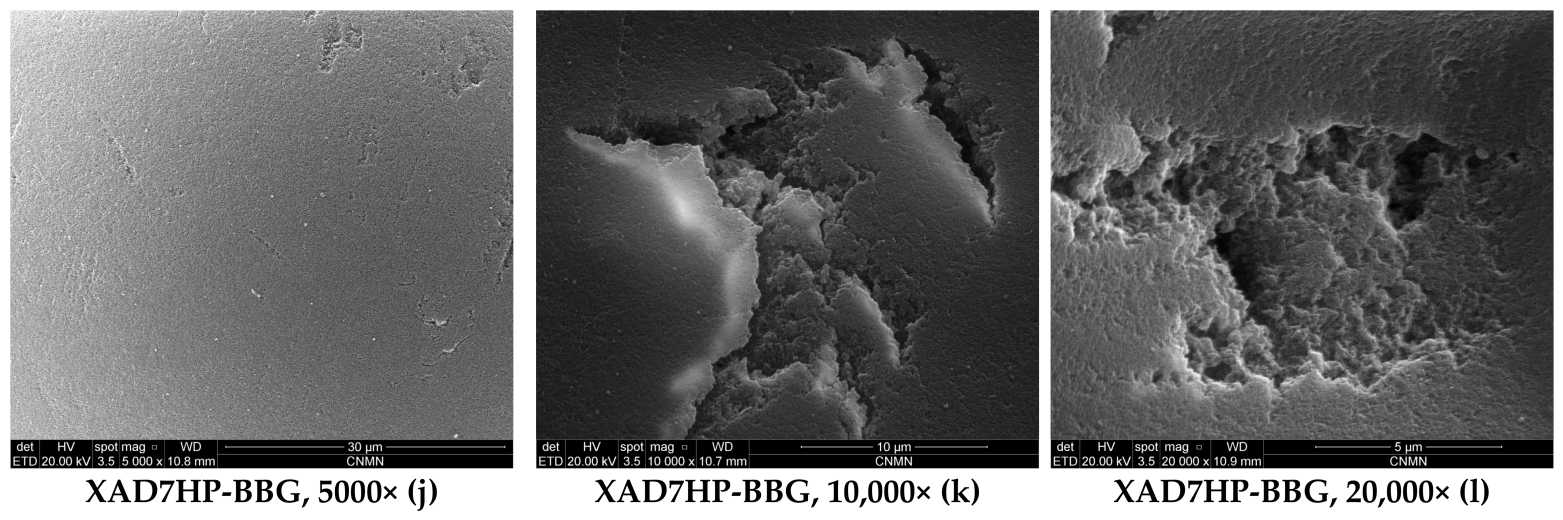
SEM analysis of XAD7HP (**a**–**f**) and XAD7HP loaded with BBG (**g**–**l**) at deferent magnifications.

To comprehensively characterize the morphological changes in the XAD7HP resin before and after BBG adsorption, SEM analysis was performed at successive magnifications (100×, 1000×, 2000×, 5000×, 10,000×, and 20,000×), allowing observation of structural evolution from the macroscopic to the nanoscale and the gradual highlighting of changes in surface, porosity, and internal architecture (Figure 9a–l).

At 100× (Figure 9a), the pristine XAD7HP resin displays a collection of nearly spherical beads with uniform size distribution and smooth external contours, consistent with the macroporous acrylicester polymer reported in previous studies [33,61,63]. The surface appears clean and free of deposits, indicating the absence of impurities or structural degradation. This morphology is typical for Amberlite resins synthesized through suspension polymerization, where spherical geometry ensures optimal hydrodynamic behavior and diffusion accessibility [62].

After BBG loading, the 100× (Figure 9g) micrograph reveals a subtle darkening of the particle surfaces and a slight reduction in visual uniformity. Although the global spherical shape is preserved, the surface appears less reflective, suggesting the formation of a thin dye layer or partial pore filling. Similar macroscopic changes have been reported for XAD7HP impregnated with organic extractants or dyes, where the adsorbate modifies the optical density and surface texture of the beads [33,37,61].

At 1000× (Figure 9b), the native XAD7HP surface shows a moderately rough texture with clearly visible pore entrances characteristic of its meso–macroporous network. These pores facilitate rapid intraparticle diffusion, a property essential for adsorption of large aromatic dyes such as BBG [74]. The polymer matrix appears intact, with no signs of swelling or collapse, consistent with the mechanical stability of cross-linked acrylic resins [62].

In contrast, the XAD7HP–BBG surface at the same magnification (Figure 9h) exhibits noticeable morphological changes. The pore openings appear partially obscured, and the surface roughness increases due to the deposition of dye molecules. The presence of BBG, a bulky triphenylmethane dye with strong π–π stacking tendencies [64,67,71], likely promotes multilayer formation or aggregation at the pore entrances. This behavior aligns with previous observations where dye adsorption leads to partial pore blockage and increased surface heterogeneity [37,65,74].

At 2000× (Figure 9c), the pristine resin reveals a well-defined porous architecture with interconnected cavities and channels. The polymeric backbone appears smooth and continuous, reflecting the typical morphology of Amberlite XAD resins [33,61]. The pore walls are clean, indicating the absence of residual monomers or manufacturing artifacts.

Upon BBG adsorption, the 2000× micrograph (Figure 9i) shows clear evidence of dye accumulation within the superficial pore network. The pore walls appear coated with a thin, irregular layer, and some cavities seem partially filled. This suggests that BBG interacts strongly with the polymer matrix, likely through hydrophobic interactions, hydrogen bonding, and π–π stacking between the aromatic rings of the dye and the polymer [53,67]. Similar deposition patterns have been reported for other anionic dyes adsorbed onto polymeric or biopolymeric matrices [65,68,75].

At 5000× (Figure 9d), the native XAD7HP surface displays a highly accessible pore system with open channels and smooth internal walls. This morphology supports efficient mass transfer and is consistent with the high adsorption capacities reported for XAD7HP in various systems [33,61,66].

After BBG loading, the 5000× image (Figure 9j), reveals significant pore obstruction. Many pore entrances appear narrowed or completely covered by dye deposits. The surface becomes more irregular, with granular or film-like structures indicative of dye accumulation. This supports the hypothesis that adsorption occurs both on the external surface and within the pore network, consistent with a mixed mechanism involving film diffusion and intraparticle diffusion [37,65,74]. The observed pore blockage is typical for large aromatic dyes, which tend to form aggregates or multilayers on polymeric adsorbents [64,68,71].

At 10,000× (Figure 9e), the pristine resin shows a finely textured surface with micro- and mesopores clearly distinguishable. The polymer matrix appears rigid and well-organized, reflecting the cross-linked nature of the acrylic backbone [62,70].

In the XAD7HP-BBG loaded sample, the 10,000× micrograph (Figure 9k) reveals a continuous dye film covering large areas of the surface. The previously visible micro-pores are now partially or completely masked. The dye layer appears compact and cohesive, suggesting strong interactions between BBG and the polymeric surface. This is consistent with the known affinity of triphenylmethane dyes for aromatic or hydrophobic domains, where π–π interactions and van der Waals forces dominate [64,67,71,74]. Such structural modifications confirm successful dye immobilization and support the kinetic evidence of rapid initial adsorption followed by slower intraparticle diffusion.

At the highest magnification 20,000× (Figure 9f), the pristine XAD7HP surface exhibits nanoscale roughness and well-defined pore edges. The polymer chains appear compact and stable, consistent with the structural integrity expected for cross-linked acrylic resins [62,70].

After BBG adsorption, the 20,000× image (Figure 9l) shows a dense, heterogeneous layer covering the nanoscale features of the resin. The dye appears to form clusters or continuous films, confirming strong surface affinity and extensive coverage. The disappearance of fine pore structures indicates that BBG penetrates deeply into the pore network and forms stable interactions with the polymer matrix. This nanoscale evidence aligns with the adsorption mechanism proposed for aromatic dyes on polymeric adsorbents, where π–π stacking and hydrophobic forces contribute to strong binding [53,67,73]. The morphological evolution across magnifications confirms that BBG adsorption on XAD7HP involves: (i) initial rapid surface deposition; (ii) progressive pore filling; (iii) formation of dye layers and aggregates; (iv) partial or complete pore obstruction; (v) strong dye–polymer interactions and (vi) a mixed diffusion mechanism, consistent with kinetic modeling. These observations are fully consistent with literature reports on dye adsorption onto polymeric resins and composite materials [37,65,66,68,69,74,75,76]. To conclude, the multiscale SEM analysis demonstrates that the porous architecture of XAD7HP governs the accessibility and distribution of BBG within the polymer matrix, highlighting the complementary roles of surface interactions and intraparticle diffusion in determining the adsorption performance. These insights strengthen the mechanistic interpretation derived from kinetic modeling and confirm the structural responsiveness of the resin during dye uptake.

#### 3.5.2. Energy-Dispersive X-Ray Spectroscopy (EDX) Analysis

**Figure 10:**
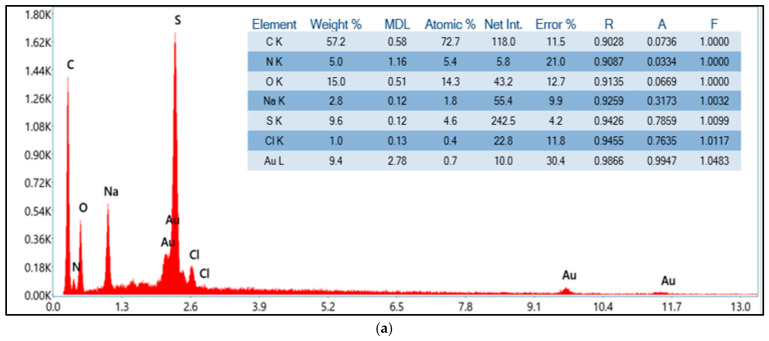

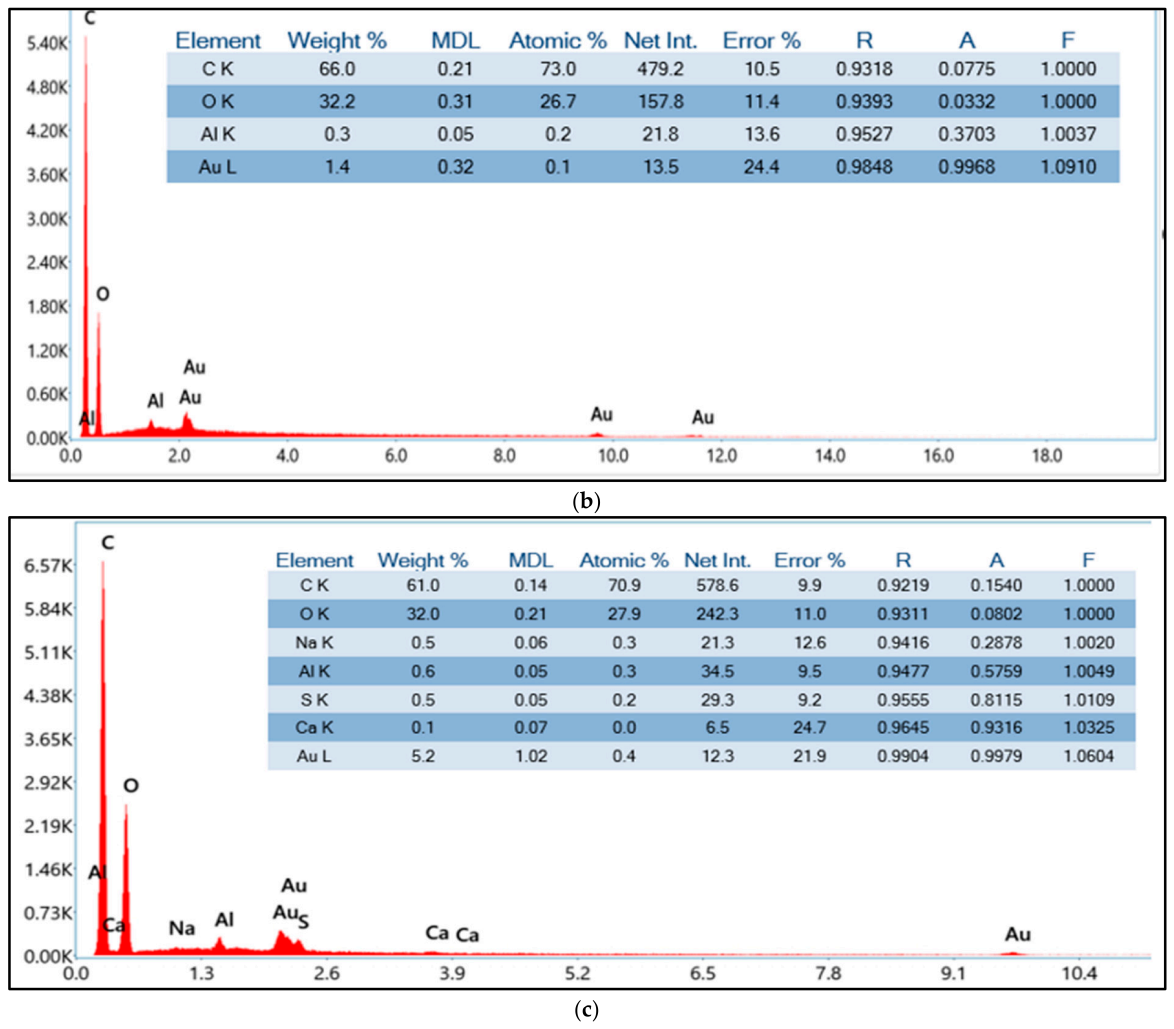
(**a**) EDS spectrum and elemental composition table for the BBG sample. (**b**) EDS spectrum and elemental composition table for the XAD7HP sample. (**c**) EDS spectrum and elemental composition table for the XAD7HP-BBG sample.

Energy-dispersive X-ray spectroscopy was employed to characterize the elemental composition of pure BBG, pristine XAD7HP, and the dye-loaded resin (XAD7HP–BBG). The comparative spectra (Figure 10a–c) provide direct elemental evidence of dye adsorption and complement the FTIR and SEM analyses.

##### EDX Spectrum of BBG

The EDX spectrum of BBG (Figure 10a) is dominated by carbon (C) and oxygen (O), consistent with the aromatic triarylmethane backbone and the oxygen-rich sulfonate substituents of the dye molecule [64,71,72]. A strong sulfur (S) signal is observed, originating from the two –SO_3_^−^ groups characteristic of disulfonated triphenylmethane dyes [64,65,73]. The presence of sodium (Na) confirms the disodium salt form of BBG. No additional inorganic elements were detected, indicating high dye purity.

##### EDX Spectrum of XAD7HP

The XAD7HP resin (Figure 10b) exhibits a simple elemental composition consisting exclusively of C and O, reflecting its poly(acrylate) structure [61,62,70]. The absence of S, Na, or other heteroatoms confirms the chemical purity of the resin and the absence of inorganic contaminants, in agreement with previous studies on XAD7HP and related acrylic adsorbents [33,61,66].

##### EDX Spectrum of XAD7HP–BBG

After adsorption, the EDX spectrum of the dye-loaded resin (Figure 10c) shows clear and unambiguous changes relative to the pristine material. Most notably, distinct S and Na peaks appear, both absent in the pure resin but characteristic of BBG. Their presence on the resin surface provides direct elemental evidence of dye uptake, consistent with previous reports where sulfur-bearing dyes or metal–extractant complexes were successfully immobilized on XAD7HP [33,37,61,63,66].

The intensity of the S signal indicates substantial deposition of sulfonate-bearing dye molecules within the superficial pore network, supporting the SEM observations of surface coverage and partial pore obstruction. A slight increase in oxygen content is also observed, attributable to the oxygen-rich sulfonate groups of BBG and to hydration water co-adsorbed during the dye–resin interaction. This is consistent with the broad O–H/N–H band at 3377 cm^−1^ identified in the FTIR spectrum of the XAD7HP–BBG complex [33,63,66,69].

##### Correlation with FTIR and SEM Findings

The EDX results strongly support the FTIR-based interpretation of the adsorption mechanism. The detection of S and Na confirms the anchoring of BBG onto the resin surface, while the preservation of the C/O polymeric matrix indicates that no chemical modification of the resin occurs—consistent with the physisorption mechanism inferred from the small (<10 cm^−1^) spectral shifts [68,69,70]. Furthermore, the localized S-rich regions observed in the EDX maps correspond to the dye aggregates visualized by SEM, validating the morphological evidence of pore blockage and surface deposition [33,61,63,66]. To conclude, EDX data provide direct elemental confirmation of BBG adsorption onto XAD7HP. The appearance of S and Na peaks in the loaded resin, combined with the increased oxygen content, demonstrates efficient dye retention and supports the proposed mechanism involving hydrogen bonding, π–π interactions, dipole–dipole forces, and hydrophobic effects [68,69,70,75,76]. These findings complement the FTIR and SEM analyses and reinforce the conclusion that XAD7HP is a highly effective adsorbent for the removal of sulfonated dyes from aqueous systems.

### 3.6. XRD Analysis

**Figure 11:**
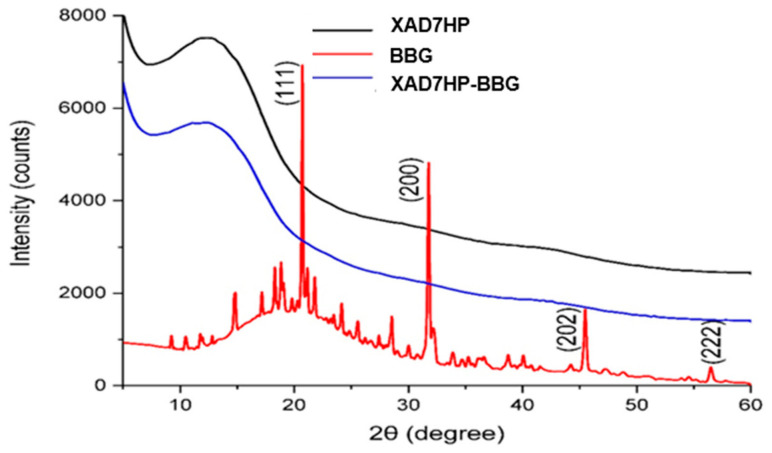
Diffractograms for XAD 7HP, BBG and XAD7HP-BBG.

The diffractograms for pure XAD7HP, BBG and XAD7HP loaded with BBG dye are presented in Figure 11. The BBG diffractogram exhibits a highly polycrystalline structure, with characteristic peaks for the Miller indices (111), (200), (202) and (222), at 2θ values of 31.79°, 45.50° and 56.55°, confirming a cubic crystal system with a lattice constant *a* = 5.631Å, in good agreement with previous reports [76,77].

The XAD 7HP exhibits a characteristic halo for amorphous materials, with a large peak in the interval 10–20°, with maximum at 13.51°, as reported before [78]. After BBG dye is adsorbed on the XAD7HP the diffractogram does not present modifications vs. pristine XAD7HP, the large amorphous halo maintaining its shape and position. At the same time, the presence of the dye’s characteristic peaks cannot be observed in the XAD7HP-BBG sample, indicating that the organic molecules were adsorbed as individually molecules and no crystalline structure is formed on the surface of the resin [78,79]. As the adsorbed BBG amount is ~10%, well over the method detection limit, the lack of long-range order in the sample XAD 7HP-BBG does confirm that the BBG is molecularly dispersed across the resin surface and trapped uniformly within the extensive internal pore network of the XAD 7HP [80]. The BBG’s amine and sulfonic moieties are generating dipole–dipole interactions with the ester/carbonyl groups along the acrylic backbone of XAD7HP. Additionally, the aliphatic chains of the cross-linked XAD7HP can interact with the aromatic rings of the BBG, pinning the dye molecules against the pore walls. As the dye molecules are spread out thinly in the pores, they cannot self-assemble or aggregate in long-range repeating lattices that generates XRD peaks.

### 3.7. TG-DSC Analysis

**Figure 12:**
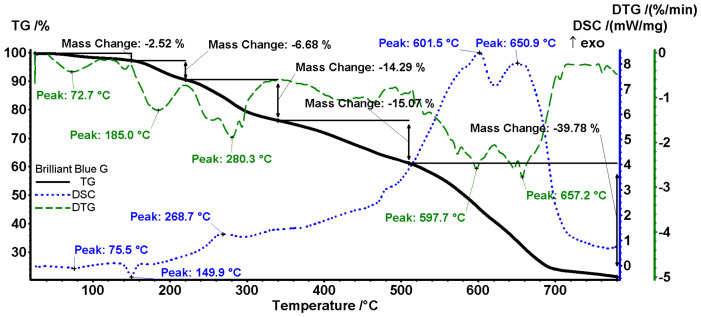
TG-DSC thermal analysis of BBG dye: degradation profiles and thermal transitions.

The thermal analysis results are presented below. The BBG dye is stable up to 150 °C. the recorded mass loss of 2.52% representing residual humidity as indicated by the weak endothermic effect from 75.5 °C. Additionally, the small peak from 149.9 °C can be assigned to melting process (Figure 12).

The thermal degradation of the dye occurs in multiple steps, partially overlapped. In the temperature interval 150–220 °C a mass loss of 6.68% is recorded, with no observable effect on the DSC curve indicating overlapping of decomposition and oxidation reactions. The oxidation reactions become dominant in the interval 220–340 °C as indicated by the exothermic effect from 268.7 °C, while the DSC curve becomes sinusoidal in the temperature interval 340–510 °C [81]. These indicate that dye molecules are fragmented and the resulting fragments are oxidized. The principal degradation step occurs after 510 °C when the carbonaceous residual mass is fully oxidized, as indicated by the strong exothermic effect on the DSC curve. The effect presents two separate peaks at 601.5 and 650.9 °C, indicating two distinct reactions. The residual mass consists of sodium sulphate.

**Figure 13:**
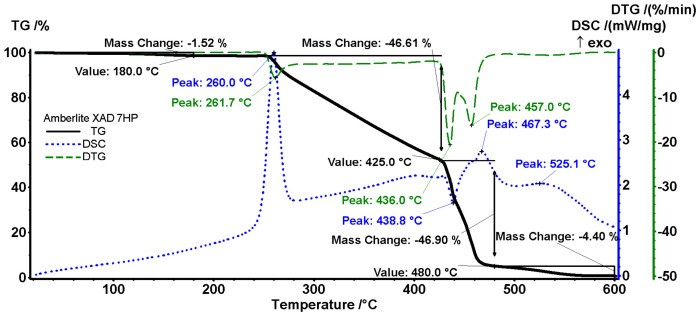
TG/DSC thermal analysis of XAD7HP: degradation profiles and thermal transitions.

The XAD7HP sample has very good thermal stability, losing a mass of 1.52% up to 180 °C, representing residual humidity [82]. The onset of the degradation process is at 256.4 °C when the XAD7HP undergoes rapid oxidation as indicated by the strong and sharp exothermic effect on the DSC curve, with maximum at 260 °C (Figure 13). This process is followed by a slower mass loss, generated by overlapping of fragmentation and oxidation reactions, that leads to a diffuse exothermic effect with maximum at 403.2 °C [81].

The mass loss recorded in the temperature interval 180–425 °C represents 46.61%. The degradation of the organic residue is completed in the temperature interval 425–480 °C, when a mass loss of 46.90% is recorded [78]. The DSC curve presents two distinct effects, one endothermic at 438.8 °C corresponding to the final fragmentation of the polymer backbone and a second exothermic effect at 467.3 °C corresponding to the oxidation of the resulting fragments. The total oxidation of the residual carbonaceous mass takes place after 480 °C, and is accompanied by a broad exothermic effect at 525.1 °C [83].

**Figure 14:**
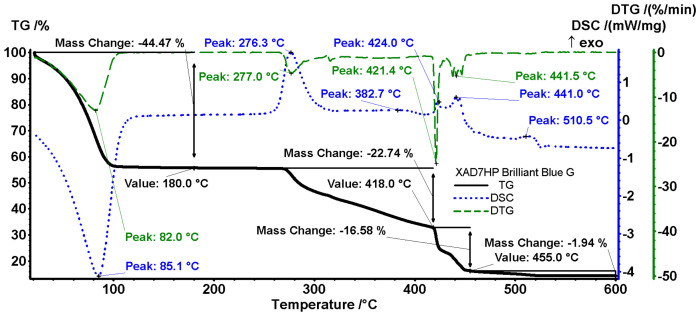
TG-DSC thermal analysis of XAD7HP-BBG d: degradation profiles and thermal transitions.

The sample XAD7HP with BBG dye presents a mass loss of 44.47% up to 180 °C, the process being associated with an endothermic effect on the DSC curve, with minimum loss at 85.1 °C (Figure 14). This process can be assigned to the elimination of the solvent molecules (water) that remained adsorbed on the XAD7HP surface and in the pores. At the same time, the DTG curve indicates that the desorption process attained the maximum speed at 82 °C.

The BBG loaded resin exhibits a similar degradation pattern as the pristine XAD7HP sample, with minor differences. After the adsorption of BBG on the XAD7HP resin, the onset of the degradation process is shifted to higher temperatures by 15.4 °C, to 271.8 °C due to the shielding generated by the dye layer, and hindered oxygen access to the XAD7HP pores. The degradation of the resin starts with an oxidation process, as indicated by the exothermic effect from 276.3 °C, and follows with a slower mass loss generated by the simultaneous fragmentation and oxidation reactions. The corresponding oxidation in the pristine resin is recorded at a lower temperature, the difference being 16.3 °C. The small exothermic effect observed for the oxidation of pristine BBG from 268.7 °C is most probably masked by the oxidation of the resin. The mass loss in the temperature interval 180–418 °C is 22.74%. The third mass loss step (16.58%), between 418 and 455 °C, is composed from two overlapped oxidation processes as indicated by the DSC exothermic effects from 424 and 441 °C. The disappearance of the endothermic effect from 438.8 °C, observable in the pristine resin, indicates a fundamental change in the mechanism or the thermal degradation, the interactions between resin and BBG promoting an oxidation reaction instead of a decomposition process. The residual carbonaceous mass is oxidized after 455 °C, the process exhibiting a broad and weak exothermic effect at 510.5 °C.

## 4. Conclusions

In this study, the adsorption of BBG onto XAD7HP was comprehensively assessed by integrating green chemistry principles with spectroscopic, kinetic, equilibrium, and morphological analyses. All stages of the process—including adsorption capacity, kinetic behavior, desorption, structural modifications of the resin, and the molecular nature of dye–polymer interactions—were systematically evaluated to demonstrate the potential of XAD7HP as an effective and sustainable material for the removal of complex dye BBG from aqueous media.

FTIR spectral analysis of XAD7HP, BBG, and the XAD7HP-BBG complex confirms the success of the adsorption process and clarifies its mechanism:

(1) The pure XAD7HP spectrum is characteristic of a crosslinked poly(methacrylate), featuring key markers at 1724 cm^−1^ [ν(C=O)] and 1141 cm^−1^ ν(C–O–C).

(2) The pure BBG spectrum exhibits the definitive markers of the triarylmethane sulfonate structure: aromatic clusters at 1575–1505 cm^−1^ and the sulfonate doublet at 1158/1109cm^−1^.

(3) The spectrum of the XAD7HP-BBG complex highlights the simultaneous presence of bands from both precursors, featuring the appearance of a new broad band at 3377 cm^−1^ (H-bonding); small shifts of the resin’s carbonyl (−4 cm^−1^) and C–O–C (+5 cm^−1^) bands; and a shift of the BBG aromatic band to 1583 cm−1 (+8 cm^−1^).

(4) The observed spectral modifications indicate an adsorption of a physical nature (physisorption), dominated by π-π, dipole–dipole, van der Waals interactions, and hydrogen bonding, without forming new covalent bonds. This conclusion aligns perfectly with thermodynamic and kinetic data reported in the literature for similar systems [33,37,64,67,70,75]. EDX analysis clearly demonstrates the efficient adsorption of BBG onto XAD7HP, as evidenced by the presence of elements characteristic of the dye (S and Na) in the XAD7HP-BBG sample, which are completely absent in the untreated resin. The high intensity of the sulfur signal confirms the anchoring of the sulfonate groups in the resin’s porous network, consistent with the FTIR changes and the deposits observed by SEM. The increase in oxygen content and the uniform distribution of the elements indicate a process of physical, homogeneous adsorption governed by hydrophobic interactions, dipole–dipole interactions, and hydrogen bonding.

Thermal analysis (TG-DSC) further demonstrated that the polymer maintains its thermal stability after BBG adsorption, with no additional decomposition stages or shifts in degradation temperatures, confirming that dye uptake does not alter the intrinsic thermal behavior of the resin.

Additionally, XRD analysis was employed to evaluate potential structural modifications of the polymeric matrix following dye uptake. The diffractograms confirmed the amorphous nature of XAD7HP and revealed that adsorption of BBG does not induce crystallinity or structural reorganization within the resin, supporting the conclusion that the interaction mechanism is governed predominantly by physisorption rather than chemical transformation.

Overall, the combined spectroscopic, morphological, structural, thermal, and kinetic evidence establishes XAD7HP as a robust, reusable, and environmentally compatible polymeric adsorbent, offering a sustainable and efficient solution for the removal of complex anionic dyes such as BBG from aqueous systems.

## Figures and Tables

**Table 1:** Kinetic modeling parameters for the adsorption process.

Morris–Weber (intraparticle diffusion)	k_id_ (mg/g min^−0.5^)	C (mg/g)	R^2^
	9.8852	17.054	0.8197
Elovich	α (mg/g∙min)	β (g/mg)	R^2^
	5.03	0.033	0.9151
PFO	k_1_ (min^−1^)	Q_e_ (mg/g)	R^2^
	0.0882	155.8	0.8895
PSO	k_2_ (g/(mg∙min))	Q_e_ (mg/g)	R^2^
	0.0002	152	0.9811

**Table 2:** Adsorption isotherm parameters obtained from the linearized adsorption models for BBG adsorption onto XAD7HP resin.

Langmuir		Temkin–Pyzhev	
Q_o_ (mg/g)	117	A (L/mg)	3.8 × 10^−2^
b (L/mg)	2.36	b_T_ (J/mol)	2.2 × 10^2^
R_L_	0.001	B	11.5
R^2^	0.9999	R^2^	0.8839
Freundlich		Dubinin–Radushkevich	
K_f_ (L/mg)	42.9	q_m_ (mg/g)	61
1/n	0.25	β (mol^2^/kJ^2^)	1 × 10^−8^
n	4.14	E (kJ/mol)	7.07
R^2^	0.5906	R^2^	0.3094

**Table 3:** FTIR Band Assignments for XAD7HP.

Wavenumber (cm^−1^)	Intensity	Assignment	Significance
2967	Medium	ν_as_(C–H) CH_3_ and CH_2_	Asymmetric C-H stretching vibration in methyl/methylene groups of the polymer chain [61,70]
2889	Weak	ν_s_(C–H) CH_2_	Symmetric C-H stretching vibration in methylene groups [70]
1724	Strong	ν(C=O) ester	Carbonyl band from the ester group (-COO); confirms the polyacrylic nature of the resin [61,62,70]
1637	Medium–Weak	ν(C=C) or δ (H_2_O)	Possible residual double bond or adsorbed water [70]
1464	Medium	δ_as_(C–H) CH_3_/δ(CH_2_)	Asymmetric angular deformation of methyl and methylene groups [62,70]
1387	Medium	δ_s_(C–H) CH_3_	Symmetric “umbrella” deformation of the methyl group [70]
1259	Medium	ν_as_(C–O–C) ester	Asymmetric stretching vibration of the ether bond in the ester group [62,70]
1141	Strongest	ν_s_(C–O–C) ester	Dominant band-symmetric C-O-C vibration; typical for poly(methacrylates) [61,62,70]
969/813/779/754	Weak–Medium	Skeletal deformations C-C, γ(C–H)	Fingerprint of the polymer backbone [70]

**Table 4:** FTIR Band Assignments for BBG.

Wavenumber (cm^−1^)	Intensity	Assignment	Significance
2973	Weak	ν(C–H) alkyl	Ethyl and benzyl groups from tertiary amino substituents (-N(C_2_H_5_)(CH_2_Ph)) [71,72].
1575	Strongest	ν(C=C) aromatic + ν_as_(SO_3_^−^)	Overlap of aromatic vibrations with asymmetric sulfonate stretching; characteristic of sulfopolychromic dyes [64,65,73].
1527	Strong	ν(C=C) + δ(N–H)	Conjugated aromatic skeletal vibrations [64,73].
1505	Strong	ν(C=C) aromatic	Cluster of bands characteristic of the trisubstituted aromatic ring of the triarylmethane skeleton [64,65].
1394	Medium	δ(C–H) CH_2_/CH_3_ + ν(C–N)	Alkyl group deformations and aliphatic C-N stretching vibrations [71,72].
1336	Medium	ν_s_(SO_3_^−^) + ν(C-N)	Symmetric vibration of the sulfonate group [65,73].
1225	Medium	ν(C–O) + ν(C–N)	Stretching vibrations of ether C-O and amine C-N bonds [64].
1158/1109	Strong	ν_as_(S=O) sulfonate	Characteristic doublet of -SO_3_^−^ groups; definitive spectroscopic marker of BBG [65,73].
1031/1009/994	Medium	ν_s_(S=O) + γ (C-H) aromatic	Symmetric S=O vibrations and in-plane aromatic C-H deformations [65,73].
906/800/ 758/691	Medium	γ(C–H) aromatic	Out-of-plane aromatic C-H deformations; typical for 1,2,4-trisubstituted rings [64,72].
615/555/421	Medium–Weak	δ(S–O) + skeletal deformations	Angular deformations of the sulfonate group and skeletal vibrations of the triarylmethane chromophore [65].

**Table 5:** Comparative Analysis of FTIR Bands for the XAD7HP-BBG.

Wavenumber (cm^−1^)	Modification	Assignment	Interpretation
3377	NEW—broad	ν(O–H)/ν(N–H)	Absent in both XAD7HP and pure BBG; emerges post-adsorption due to hydrogen bonding and hydration water [63,66,69]
2972	–	ν(C–H) alkyl	Present in both precursors; overlap remains unmodified
1720 (←1724)	Shifted − 4 cm^−1^	ν(C=O) ester	Red shift compared to pure XAD7HP; indicates interaction with BBG sulfonate groups [61,66]
1636 (←1637)	Increased intensity	ν(C=C) aromatic/δ(H_2_O)	Enhanced by contributions from BBG aromatic rings [33,66]
1583 (←1575)	New vs. XAD7HP	ν(C=C) aromatic BBG	Blue-shifted from 1575 cm^−1^; exclusively originates from BBG, confirming dye presence on the resin [33,64,66]
1465 (←1464)	–	δ CH_2_/CH_3_)	No significant variation [61,70]
1388 (←1387)	–	δ_s_(CH_3_)	No significant variation [61,70]
1259	Conserved	ν_as_(C–O–C) ester	Unchanged; no substantial modification [61,70]
1146 (←1141)	Shifted + 5 cm^−1^	ν_s_(C–O–C) ester	Blue shift; indicates overlap with BBG ν(S=O) [61,63,65,66]
959	Modified	δ(C–H) skeleton	Slight change relative to XAD7HP bands at 969 cm^−1^ [61,66]
749	Conserved	γ(C–H) aromatic	BBG aromatic bands are retained with minimal shifts [64]

(Note: Rows with major spectral changes relative to pure precursors are bolded/indicated by specific modifications).

## Data Availability

The original contributions presented in this study are included in the article. Further inquiries can be directed to the corresponding author.

## Supplementary Materials

The following supporting information can be downloaded at: https://www.mdpi.com/article/10.3390/polym18141763/s1, Figure S1: Adsorption isotherm model fitting: (a) Freundlich, (b) Temkin, and (c) Dubinin–Radushkevich; Figure S2: Kinetic model fitting: Weber–Morrisintraparticle diffusion model (a), Elovich model (b), PFO model (c), and PSO model (d); Figure S3: Overlapped FTIR spectra of XAD7HP, BBG, and XAD7HP–BBG.
