# Supplementary material for: Integrating Green Chemistry and Analytical Spectroscopy for Brilliant Blue G Removal Using Amberlite XAD7HP Resin
Source: Polymers (Basel). 2026 Jul 19;18(14):1763. doi: 10.3390/polym18141763 (PMC13416592; doi:10.3390/polym18141763)
Supplement: Supplementary file 1 [file polymers-18-01763-s001.zip › polymers-4404210-supplementary.pdf]

polymers

MDPI

Supplementary Material

Integrating Green Chemistry and Analytical Spectroscopy for Brilliant Blue G Removal Using Amberlite XAD7HP resin

Nicoleta Mirela Marin[1,2,3,*], Toma Galaon[1,2] Adriana Mariana Borş[4], Ludmila Motelica[5,6], Otilia Ruxandra Radacina[3], Marian Rascov[3] and Ovidiu Oprea[6,7,8]

[1] National Research and Development Institute for Industrial Ecology ECOIND, Street Podu Dambovitei no. 57-73, District 6, 060652 Bucharest, Romania; tomagalaon@yahoo.com (T.G.)
[2] Department of Analytical and Physical Chemistry, University of Bucharest, 4-12 Regina Elisabeta Bd., 030018 Bucharest, Romania (N.M.M.)
[3] Department of Oxide Materials Science and Engineering, National University of Science and Technology Politehnica Bucharest, 1–7 Gh. Polizu, 060042 Bucharest, Romania; otiliaradacina@yahoo.com (O.R.R); marian.rascov@yahoo.com (M.R),
[4] National Institute for R&D for Optoelectronics - Subsidiary, Research Institute for Hydraulics and Pneumatics—INOE 2000-IHP, 040558 Bucharest, Romania; bors.ihp@fluidas.ro (A.M.B.)
[5] Research Center for Advanced Materials, Products and Processes, National University of Science and Technology POLITEHNICA Bucharest, Splaiul Independentei 313, 060042 Bucharest, Romania; ludmila.motelica@upb.ro (L.M.)
[6] National Centre for Micro- and Nanomaterials, National University of Science and Technology Politehnica Bucharest, 313 Independence Boulevard, 060042 Bucharest, Romania; ovidiu.oprea@upb.ro (O.C.O.),
[7] Academy of Romanian Scientists, 3 Ilfov St., 050045 Bucharest, Romania
[8] Faculty of Chemical Engineering and Biotechnologies, National University of Science and Technology POLITEHNICA Bucharest, Gh. Polizu 1-7, 011061 Bucharest, Romania
*Correspondence: nicoleta.marin@incdecoind.ro

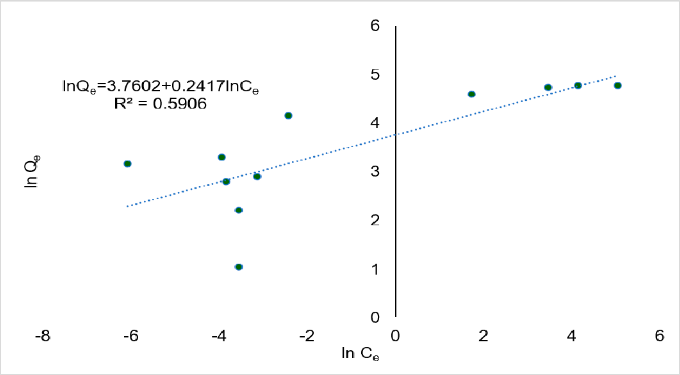

(a)

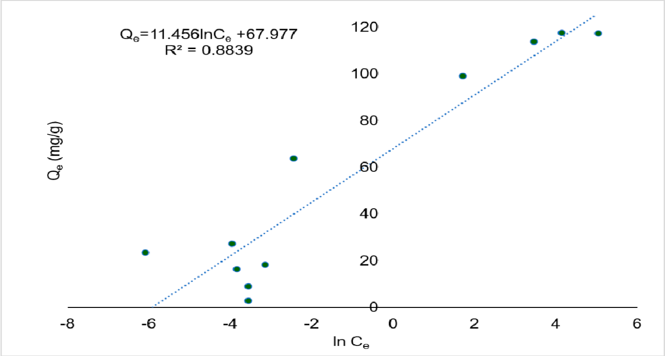

(b)

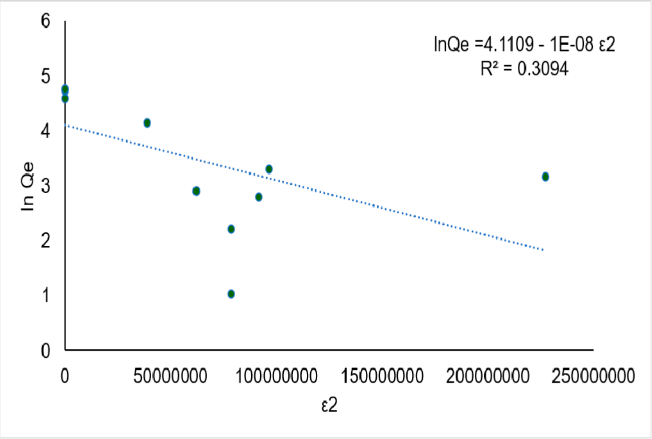

Figure S1. Adsorption isotherm model fitting: (a) Freundlich, (b) Temkin, and (c) Dubinin–Radushkevich.

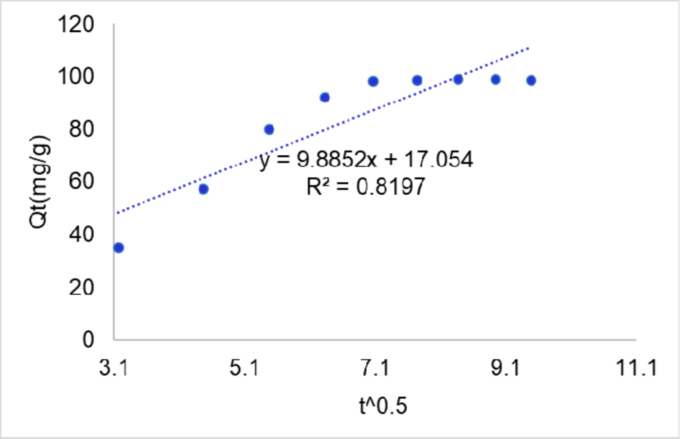

(a)

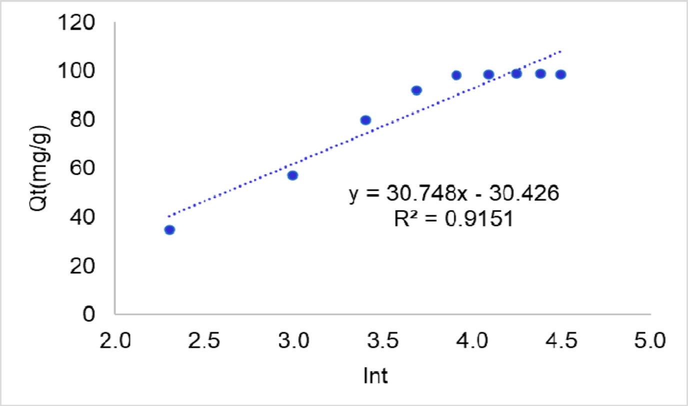

(b)

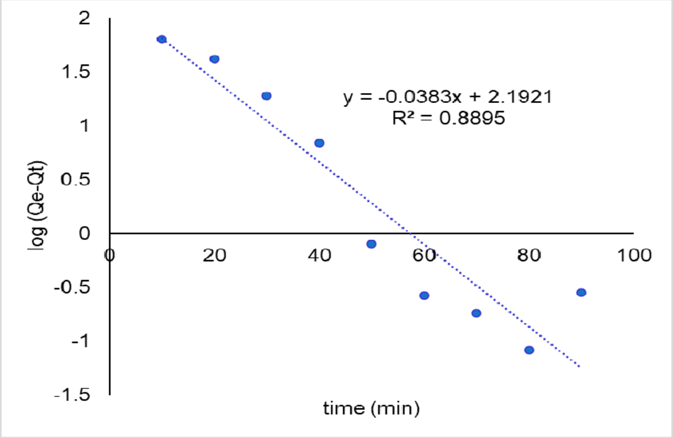

(c)

https://doi.org/10.3390/polym18141763

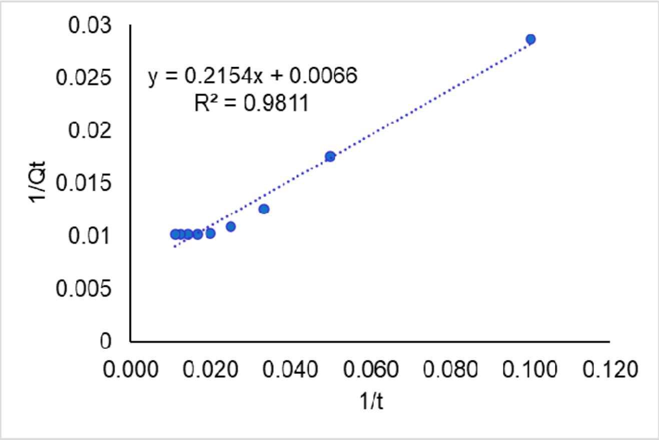

(d)

Figure S2. Kinetic model fitting: Weber–Morrisintraparticle diffusion model (a), Elovich model (b), PFO model (c), and PSO model (d)

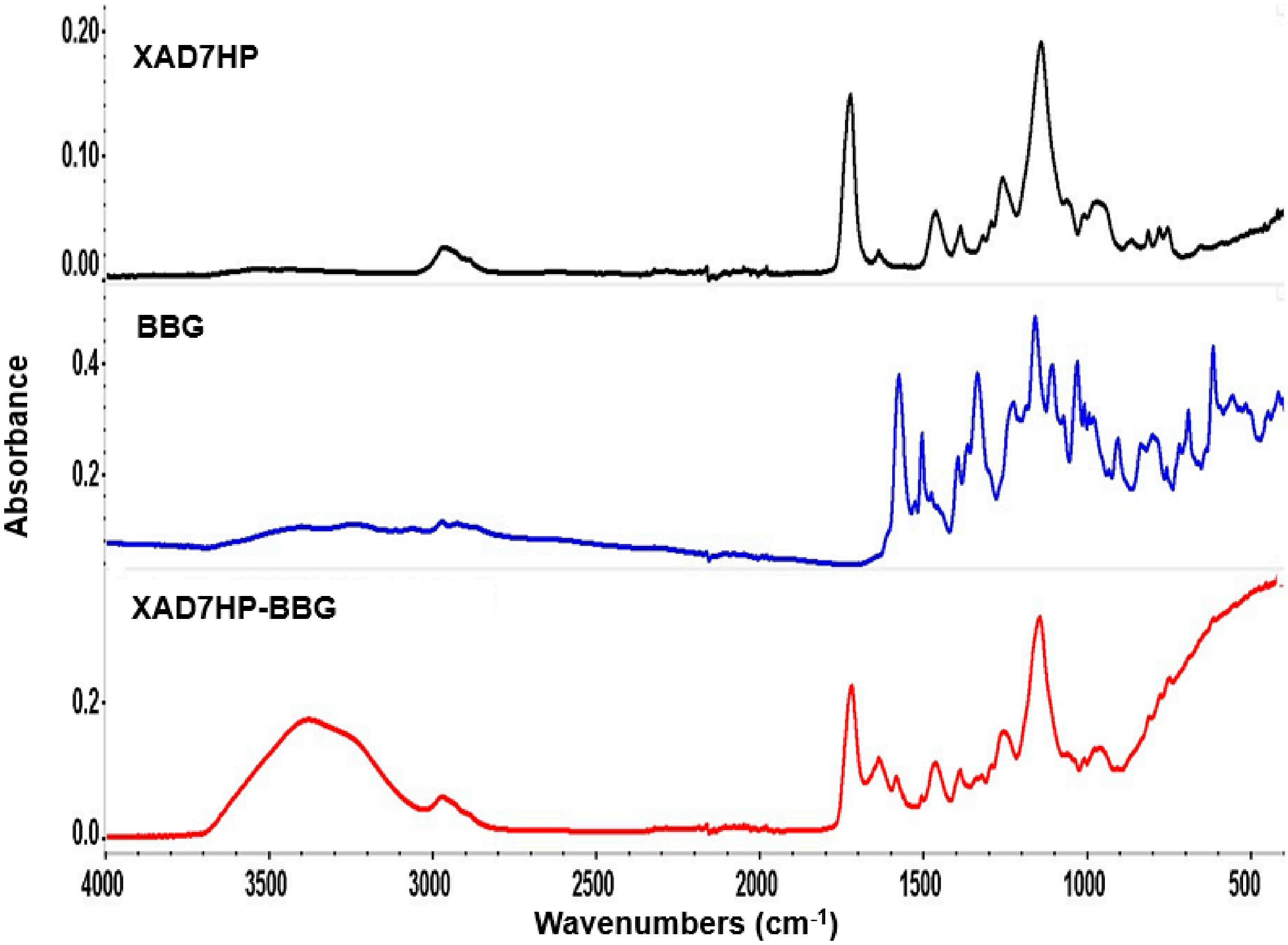

Figure S3. Overlapped FTIR spectra of XAD7HP, BBG, and XAD7HP–BBG.

https://doi.org/10.3390/polym18141763
